# Synthesis and Characterization of Gum Rosin Microspheres Containing Zinc Phthalocyanine Prepared by the Electrospraying Method

**DOI:** 10.3390/polym18212781

**Published:** 2026-11-07

**Authors:** Cristina Pavon, Cristina Mellinas, María Carmen Garrigós, Alfonso Jimenez, Juan López-Martínez

**Affiliations:** 1 Instituto Universitario de Investigación de Tecnología de los Materiales, Univesitat Politècnica de València, 03801 Alcoy-Alicante, Spain; cripava1@epsa.upv.es (C.P.); jlopezm@mcm.upv.es (J.L.-M.); 2 Departamento de Química Analítica, Nutrición y Bromatología, Universidad de Alicante, 03690 San Vicente del Raspeig, Spain; cristina.mellinas@ua.es (C.M.); mc.garrigos@ua.es (M.C.G.)

**Keywords:** gum rosin, microspheres, zinc phthalocyanine, encapsulation, electrospraying

## Abstract

Gum rosin (GR) is a natural and renewable polymer resin obtained from pine trees with potential in medication delivery applications. This work uses electrospraying to obtain and characterize GR microspheres (eGR) containing zinc phthalocyanine (ZnPc), a synthetic blue-green macrocyclic chemical with photosensitive properties. Electrospraying effectively created uniform microspheres with high encapsulation efficiency. A ZnPc release profile was performed, and it was observed that it quickly evolved during the first 24 h, reaching 0.438 mg per 100 g of sample and peaking at 0.868 mg per 100 g after 72 h. The subsequent decline is presumably due to the high hydrophobicity of free ZnPc, leading to its precipitation or adsorption onto the container. Nuclear magnetic resonance (NMR) analysis demonstrated the interaction between ZnPc and GR, confirming the successful formation of microspheres. Zeta potential analysis indicated that both eGR and eGR-ZnPc microspheres had negatively charged surfaces, likely due to anion adsorption or the presence of functional groups. The initial zeta potential values were −80.6 ± 3.3 mV for eGR and −74.1 ± 5.6 mV for eGR-ZnPc, reflecting the high stability of microspheres. However, stability decreased after 120 h due to particle aggregation, with a 70% drop in zeta potential after 360 h. No significant differences were observed between formulations during stability tests.

## 1. Introduction

Gum rosin (GR) is a natural, renewable resin obtained from the pine tree. It is composed predominantly of resin acids, including abietic acid and related diterpenoid compounds. Film-forming ability, hydrophobicity, biodegradability, and chemical versatility have made GR and its derivatives attractive materials for pharmaceutical, biomedical, environmental, and electronic applications [1,2,3,4]. Rosin-based materials have been investigated as film-forming and coating agents in pharmaceutical applications, particularly for sustained drug release. At the same time, more recent studies have expanded their use toward nanocarriers and other advanced drug-delivery systems [1,4,5]. These characteristics, together with the possibility of chemically modifying rosin or combining it with other polymers, make GR a promising bio-based matrix for the incorporation and controlled delivery of active compounds [1,5].

The development of gum rosin-based microspheres represents an attractive strategy for exploiting these properties in drug-delivery applications. Early studies demonstrated that rosin derivatives could be used to prepare microcapsules capable of controlling the release of incorporated drugs [4]. More recently, GR has been incorporated into polymeric micro- and nanostructures for the delivery of active pharmaceutical compounds. For example, 6-thioguanine-loaded gum rosin/poly(ethylene oxide) microspheres were reported as controlled-release systems, with the active compound incorporated into the polymeric matrix and the resulting microspheres exhibiting a spherical morphology [6]. Similarly, rosin-ester-based polymeric nanocarriers loaded with 5-fluorouracil and carmofur were proposed for anticancer drug delivery, demonstrating the potential of rosin-derived matrices as carriers for pharmacologically active compounds [7]. These studies support the suitability of rosin-based materials as matrices for incorporating and controlling the delivery of active substances.

While unmodified GR has been explored in various pharmaceutical applications, conventional formulation methods present significant drawbacks for photosensitizer delivery. As summarized in Table 1, macroscopic GR matrices often require thermal processing, while conventional GR microparticles rely on multi-step emulsions requiring surfactants. The unique scientific feature of our electrosprayed GR (eGR) platform is its ability to produce highly uniform, surfactant-free microspheres in a single step at room temperature. This approach circumvents the limitations of previous GR systems, preserving the physicochemical integrity of the hydrophobic ZnPc while providing the high surface-area-to-volume ratio necessary for a controlled biphasic release.

Among the techniques available for producing particulate drug-delivery systems, electrospraying is a versatile electrohydrodynamic process that enables the preparation of micro- and nanoparticles from polymeric solutions or dispersions [10,11,12]. This technique offers several advantages, including relatively simple processing, low material consumption, reproducibility, and the ability to tune particle size and morphology through formulation and processing parameters such as polymer concentration, solution properties, applied voltage, flow rate, and collector distance [10,11]. Electrospraying can be used to incorporate active compounds into polymeric particles, offering opportunities for controlled delivery and protection of poorly soluble or sensitive compounds [13,14,15]. However, particle morphology and size distribution are highly dependent on the physico-chemical properties of the polymer matrix and the operating conditions; therefore, uniform or monodisperse particles should not be considered an intrinsic characteristic of the technique [14]. The suitability of electrospraying for GR processing has also been demonstrated by Pavon et al., who reported the preparation of GR microspheres by electrospraying and their subsequent incorporation into poly(ε-caprolactone) bilayer films [16]. These authors demonstrated that processing parameters could be adjusted to obtain GR microspheres with controlled morphology and suitable particle size. In a subsequent study, Pavon et al. further demonstrated that electrosprayed GR microspheres can serve as carriers for inorganic nanoparticles, including silver nanoparticles and halloysite nanotubes [8]. Although the particle-size distribution of the microspheres was relatively broad, their incorporation was successfully observed within the GR particles, supporting the feasibility of using electrospraying to generate functional gum rosin-based microparticles. These findings provide a direct technological basis for developing GR microspheres containing additional functional compounds.

On the other hand, zinc phthalocyanine (ZnPc) is a metal phthalocyanine with unique photophysical and photochemical properties that has attracted considerable attention as a photosensitizer for photodynamic therapy (PDT) [17,18]. In PDT, the photosensitizer is activated by light at an appropriate wavelength. It can transfer energy to molecular oxygen, generating reactive oxygen species (ROS), particularly singlet oxygen, which can induce oxidative damage to biological targets [17,18]. Recent studies have reported the photodynamic potential of ZnPc and related zinc(II) phthalocyanines, including their ability to generate ROS and induce light-dependent biological effects [9,19]. Despite these interesting properties, the biomedical application of ZnPc is limited by its poor aqueous solubility and strong tendency to aggregate. Aggregation can modify the photophysical behavior of phthalocyanines and reduce the availability of the photoactive species, thereby limiting their effectiveness as photosensitizers [20]. Consequently, the incorporation of phthalocyanines into appropriate delivery systems has become an important strategy for improving their dispersion, stability, biodistribution, and photodynamic performance.

A wide range of delivery systems, including liposomes, polymeric micelles, polymeric nanoparticles, and other nanostructured carriers, have therefore been investigated for phthalocyanine-based PDT [16,17,18,19,20,21,22]. Recent studies demonstrated the relevance of polymeric carriers for ZnPc delivery. For example, ZnPc has been efficiently encapsulated in chitosan micelles, and the polymer-to-ZnPc ratio was shown to influence the stability and phototoxicity of the resulting formulations [19]. ZnPc-containing polymeric nanocarriers have been investigated as pH-responsive systems for photodynamic cancer treatment, with encapsulation within the polymeric carrier improving photosensitizer delivery and promoting ROS generation under irradiation [23]. Other recent approaches have used polymeric or self-assembled nanocarriers to encapsulate hydrophobic ZnPc and investigate the relationship between carrier structure, photosensitizer organization, and PDT performance [24]. These studies demonstrated that incorporating ZnPc into polymeric structures is an adequate research strategy for overcoming limitations associated with their intrinsic physicochemical properties. The incorporation of ZnPc into electrospun polymeric structures has also been reported. Rossin et al. prepared ZnPc-containing PBAT/PLA fibers via electrospinning. They demonstrated the feasibility of incorporating the photosensitizer into a polymeric fibrous matrix while preserving its photoactive functionality [13]. All these studies highlight the versatility of polymer matrices in incorporating and delivering phthalocyanine-based photosensitizers. Nevertheless, most reported ZnPc delivery systems rely on nanoscale carriers or polymeric fibers, whereas the use of bio-based rosin-derived microspheres as ZnPc carriers remains comparatively unexplored.

While previous studies successfully encapsulated ZnPc in synthetic nanocarriers [23,24], chitosan micelles [19], or electrospun fibers [13], these approaches often require complex syntheses and/or produce fibrous morphologies that limit localized injection. The core innovation of this work is the use of gum rosin—a renewable, natural bio-resin—to produce standalone spherical microcarriers via a single-step electrospraying process. Compared with established biopolymeric systems, such as PLGA or functionalized chitosan, the proposed electrosprayed gum rosin platform offers a more sustainable, cost-effective, and simpler formulation strategy. This eGR-ZnPc system leverages the intrinsic hydrophobicity of natural rosin to encapsulate the photosensitizer, offering a fully bio-based alternative for localized delivery.

In this study, ZnPc-loaded gum rosin microspheres were prepared by electrospraying and characterized by their physicochemical properties, encapsulation performance, release behavior, and colloidal stability. Particular emphasis was placed on incorporating ZnPc into the GR matrix, its influence on microsphere morphology and surface properties, and the interactions between the photosensitizer and the rosin matrix. The release profile of ZnPc and the temporal evolution of microsphere stability were further investigated to assess the potential of electrosprayed GR as a bio-based matrix for photosensitizer delivery.

## 2. Materials and Methods

### 2.1. Materials

Gum rosin (GR) commercial-grade (CAS Number: 8050-09-7) was supplied by Sigma-Aldrich (Mostoles, Spain) to ensure baseline consistency in purity and acid number. GR is characterized by a molecular weight of 302 g/mol, a softening point of 66.5 °C, and an acid number of 167. Chloroform was supplied by Panreac (Barcelona, Spain) with a density >1.48 g/cm^3^ at 20 °C and 99.8% purity. Zinc phthalocyanine (ZnPc) was also supplied by Sigma-Aldrich. To overcome any inherent minor compositional variation typical of natural resins, the formulation parameters (GR concentration and solvent ratios) were strictly standardized to ensure highly reproducible microsphere formation.

### 2.2. Electrospraying Process

A GR/chloroform 45% (*w*/*v*) solution was prepared and continuously stirred at room temperature for 24 h. Furthermore, ZnPc, previously dissolved in dimethylformamide (DMF) (6.35 × 10^−4^ M), was added to the GR solution and agitated for 2 h. This solution was subjected to electrospraying with the following parameters: target distance to the collector 15 cm, applied voltage 10 kV (positive and negative voltages of 5 kV and −5 kV, respectively), flow rate 5 µL/min, and electrospraying time 4 h to obtain gum rosin microspheres containing ZnPc (eGR-ZnPc). These specific electrospraying parameters were adopted from a previous optimization study on pure GR microspheres [16], as they guarantee a stable Taylor cone, and ensure the highly dilute addition of ZnPc did not alter the optimal jetting conditions. A Higher Pressure Programmable Single Syringe Pump NE-1010 (Shropshire, UK) was used to deliver the solution, and power was supplied by a Genvolt 7xx30 series unit (Shropshire, UK). A scheme of the experimental work for electrospraying GR/ZnPc solutions is shown in Figure 1.

### 2.3. Field Emission Scanning Microscopy (FESEM)

The microspheres were deposited onto aluminum foil for 4 h, and their morphology was analyzed by field-emission scanning electron microscopy (FESEM) using a Zeiss Ultra 55 microscope (Jena, Germany) at 1 kV.

### 2.4. Fourier Transform Infrared Spectroscopy (FTIR)

FTIR spectra were recorded using the KBr-disc transmission technique. To ensure accurate analysis and comparison of the characteristic absorption bands, the spectral baselines were corrected and data were normalized using the PerkinElmer Spectrum IR software (version 10.6.0) before plotting.

### 2.5. Ultraviolet-Visible Spectroscopic Analysis (UV-Vis)

The UV–Vis spectroscopic analyses were carried out on a Cary 100 UV–Vis spectrophotometer by Agilent Technologies (Barcelona, Spain). All measurements were performed over a scanning range of 800 to 200 nm with a 1 nm step size. The concentration of ZnPc deposited in e-GR was determined by the Lambert-Beer Law (Equation (1)):

(1) A=ε·c·𝓁,

where A is the absorbance (measured by the UV-Vis spectrophotometer), ε is the molar absorptivity (a constant specific to the substance at a given wavelength), c is the concentration of the solution, and 𝓁 is the path length of the cuvette (typically 1 cm). ε was calculated using a calibration curve from the slope of the linear relationship between absorbance and concentration.

### 2.6. Nuclear Magnetic Resonance (NMR) Analysis

For the 1H-NMR assay, approximately 20 mg of each material (ZnPC, GR, and eGR-ZnPc) was used and dissolved in 0.6 mL of deuterated chloroform. The liquid-state NMR experiments were performed using a 400 MHz NMR spectrometer (BRUKER AVANCE NEO, Billerica, MA, USA).

### 2.7. Stability Evaluation

The stability of microcapsules was evaluated under refrigerated conditions. Dispersions of eGR-ZnPc (0.2 mg/mL) in deionized water with 1 wt% of sodium dodecyl sulfate (SDS) were prepared and maintained at 4 °C for 15 days. Z-potential values were measured with a DLS Zetasizer Nano-ZS (Malvern Instruments, Malvern, UK). Measurements were performed, in triplicate, using a disposable folded capillary cell and polystyrene cuvettes.

### 2.8. Release of the Active Compound

The use of 0.9 wt% NaCl as a simulant in medical research is based on its isotonicity, which makes it useful for simulating various physiological conditions, particularly in studies involving intravenous fluids, tissue hydration, or drug delivery systems. This is the reason why it has been selected to evaluate the ZnPc release in GR-based microspheres. 2 mg of active microcapsules were dissolved into 10 mL of 0.9 wt% NaCl with 1 wt% of SDS and were kept at 4 °C for 10 days with measurement points at 1, 2, 3, 5, and 10 days. Samples were filtered to remove solid particles, and the liquid was analyzed by obtaining its UV–Vis spectra. ZnPc was used to obtain the calibration curve (0.84–4.63 mg/kg, r^2^ = 0.9994). All tests were performed in triplicate [25].

## 3. Results and Discussion

### 3.1. Field Emission Scanning Microscopy (FESEM)

Figure 2 shows the FESEM images of e-GR and e-GR-ZnPc, along with histograms of their diameter distributions. The average diameter of e-GR microspheres was approximately 6.4 ± 1.1 µm, while the e-GR-ZnPc microspheres had an average diameter of 20.9 ± 3.3 µm. This significant increase in the average diameter of e-GR-ZnPc microspheres compared to those from e-GR strongly suggests the successful incorporation of ZnPc into the polymer matrix. This substantial increase in diameter cannot be attributed solely to the physical mass of the incorporated ZnPc. Instead, it suggests that the inclusion of ZnPc altered the microscopic electrospraying dynamics resulting in additional increase in the average diameter of microspheres. As supported by 1H NMR analysis, intermolecular interactions between GR and ZnPc likely increase cohesive forces within droplets, thereby resisting secondary Coulombic fission reactions. Additionally, the hydrophobic ZnPc macrocycles may act as early nucleation centers, altering the kinetics of solvent evaporation and causing the droplets to precipitate and solidify at a larger diameter.

Furthermore, the FESEM images revealed clustering and aggregation in the e-GR-ZnPc microspheres, due to the ZnPc incorporation. However, it is important to note that when dispersed in a solution, the microspheres separated and behaved as individual entities. This suggests that the microspheres remained stable and evenly distributed in a liquid environment, and that the aggregation observed in the FESEM micrographs likely results from solvent evaporation during drying after electrospraying. This dispersed state in liquid media was further supported by the high initial zeta potential values (−74.1 ± 5.6 mV, see Section 3.5), which provide quantitative evidence of strong electrostatic repulsion between individual microspheres, preventing their aggregation in solution.

### 3.2. Fourier Transform Infrared Spectroscopy (FTIR)

FTIR spectroscopy was used to confirm the interaction between ZnPc and GR microspheres (e-GR). Figure 3a shows the FTIR spectra of e-GR, ZnPc, and e-GR-ZnPc, highlighting their characteristic bands. The FTIR spectrum of e-GR displays absorption bands corresponding to the main components of GR. For example, a shoulder at 3336 cm^−1^ corresponds to the carboxyl group’s stretching vibration ν(O-H) (-COOH) [26]. A double peak at 2944 cm^−1^, with a shoulder at 2872 cm^−1^, reflects strong C–H stretching vibrations of methyl and methylene groups, typical of the hydrocarbon structure in diterpenes [27]. Additionally, a broad double-structured band at 2652 and 2536 cm^−1^ corresponds to overtone stretching of the carboxyl group [27]. The carbonyl stretching ν(C=O) of carboxylic acid is observed at 1694 cm^−1^ [28,29], with characteristic bands at 1278 cm^−1^ (C-O-C) and 712 cm^−1^, identifying gum rosin [27]. The absorption bands between 1200 cm^−1^ and 800 cm^−1^ are typical of diterpenic resins [27].

In the ZnPc spectrum, the peak at 634 cm^−1^ corresponds to the C–C macrocycle ring deformation, while those at 752 and 780 cm^−1^ are attributed to C–N stretching [30]. The 1060 cm^−1^ peak reflects C–N stretching in pyrrole, and those at 918, 878, and 726 cm^−1^ indicate C–H out-of-plane deformations [30]. Peaks at 1113 and 1083 cm^−1^ are due to C–H bending and in-plane deformations, while those at 1284, 1160, and 1060 cm^−1^ correspond to C–N stretching in isoindole and pyrrole groups [30]. The peak at 1487 cm^−1^ is due to C–H aryl, and those at 1410 and 1335 cm^−1^ correspond to C–C stretching in isoindole, while the band at 1608 cm^−1^ represents C–C stretching in pyrrole, and those at 2923 and 2856 cm^−1^ are assigned to C–H asymmetric stretching in alkyl groups. Bands at 3074 and 3048 cm^−1^ correspond to C–H stretching in the aromatic ring, and the broad band at 3423 cm^−1^ is attributed to OH vibrations from adsorbed water [30].

By comparing the spectra from e-GR, e-GR-ZnPc, and ZnPc (Figure 3a), it is observed that most of the absorption bands of e-GR overlap with those of ZnPc. Additionally, due to the low concentration of ZnPc, its characteristic bands show low intensity. However, distinct bands at 3423, 1160, 634, 726, and 780 cm^−1^, along with a shoulder at 1608 cm^−1^, suggest successful deposition or interaction between GR microspheres and ZnPc molecules. To fully elucidate the nature of intermolecular interactions and overcome the significant overlap of characteristic absorptions, a difference FTIR spectrum was performed by subtracting the e-GR spectrum from the e-GR-ZnPc. The resulting difference spectrum (Figure 3b) revealed a prominent positive absorbance feature at 3420 cm^−1^ and significant derivative-like shifts in the carbonyl and macrocycle region (1800–1600 cm^−1^). These distinct non-zero features confirm that the incorporation of ZnPc is not merely a physical mixture, but involves distinct intermolecular interactions, likely hydrogen bonding or dipole–dipole interactions, between the photosensitizer and the rosin matrix.

### 3.3. Ultraviolet-Visible Spectroscopic Analysis (UV-Vis)

Figure 4 shows the UV-Vis spectra of e-GR, ZnPc, and their combination (e-GR-ZnPc). The optical properties of phthalocyanine molecules and their derivatives arise from their unique ring structure, which produces two distinct energy bands: the B-band (γ or Soret band) and the Q-band (α band in porphyrins) [31,32]. ZnPc exhibits two primary absorption bands in the visible/near-UV spectrum: the B-band, located at approximately 360 nm, and the Q-band at around 669 nm, corresponding to the first π–π* transition on the phthalocyanine macrocycle [26]. Additionally, low-intensity vibrational features appear around 600 nm and are associated with vibrational components [26].

In contrast, e-GR displays an intense peak centered at 271 nm, attributed to abietic acid, whereas oxides exhibit a less well-defined absorbance in the same region [23]. Notably, the spectra of e-GR-ZnPc retain the characteristic absorption bands of both GR and ZnPc, confirming the successful incorporation of ZnPc into the GR microspheres and indicating proper deposition and integration of the phthalocyanine onto the microsphere matrix, resulting in an effective combination of these two components.

By using the known ZnPc concentration from the UV-Vis analysis (6.36 × 10^−7^ M), the linear relationship between absorbance and concentration from the calibration curve can be determined as:

A=281800·c·𝓁

where ε, the molar absorptivity, is 281,800 (M cm)^−1^ [33]. The concentration of ZnPc deposited in e-GR for the different e-GR-ZnPc combinations used in this study is shown in Table 2.

These results highlighted the direct relationship between sample mass, absorbance, and ZnPc concentration in e-GR microspheres, while also considering high ZnPc dispersion in the microspheres and matrix effects that may influence the results, particularly the linearity of the curve. Two main hypotheses could explain these results: (1) ZnPc concentration increases with the mass of e-GR microspheres, and (2) the absorbance-concentration relationship is influenced by interactions between ZnPc and microspheres, suggesting that the system behavior is more complex than expected, drawing attention to the need for further investigation into experimental conditions and interactions between both components.

### 3.4. Nuclear Magnetic Resonance (NMR) Analysis

The analysis of the NMR spectra from metallo-phthalocyanine (MPc) complexes, including ZnPc, is based on the shift in the 1H signal downfield in response to a stronger ring current. The 1H-NMR spectra for ZnPc, eGR, and eGR-ZnPc are shown in Figure 5, with particular emphasis on the region between 1–3 ppm, where significant differences among the spectra were observed.

The NMR analysis of GR microspheres has been little studied by other researchers [34]. In this study, the 1H-NMR spectrum of e-GR is shown in Figure 5a. A clear peak at around 2.1–2.2 ppm was observed, corresponding to the main -OH protons in the GR structure. Other prominent peaks were observed around 1.2 ppm, corresponding to functional groups that are likely to form hydrogen bonds [34]. When examining the microsphere spectrum, some interactions between ZnPc and the polymer matrix are suggested (Figure 5c). The peaks observed in the 2.0–3.0 ppm region correspond exclusively to the cycloaliphatic methylene and methine protons of the GR matrix. Upon incorporation of ZnPc, spectral variations were observed in this region. Since unsubstituted ZnPc lacks aliphatic protons, these variations are attributed to the shielding and deshielding effects exerted by the large, highly aromatic ZnPc macrocycle on the surrounding GR molecules. Additionally, a characteristic –OH peak was observed at 2.1 ppm, as expected from the GR spectrum. This alteration of the local magnetic environment of the rosin protons serves as strong evidence of physical and intermolecular interactions between the GR matrix and the encapsulated ZnPc.

### 3.5. Stability Evaluations

Zeta potential is a critical parameter for characterizing the stability of particles suspended in a solution, such as colloids or nanoparticles. It measures the electric potential existing at the boundary between charged particles and the surrounding liquid. Particles with high zeta potential exhibit strong electrostatic repulsion, preventing their aggregation or clot formation and maintaining their dispersed state in the solution [35]. Generally, a zeta potential value exceeding ±60 mV is considered a reliable indicator of excellent particle stabilization within the liquid medium. Furthermore, a negative zeta potential value suggests that the surface of the suspended particles carries a negative charge. This can be due to the adsorption of negatively charged ions (anions) onto the particle surface, or to the nature of the functional groups present on the surface [36]. As a natural resin, GR contains various functional groups (e.g., carboxyl groups) that can ionize or interact with ions in solution, leading to the generation of surface negative charges [37]. The negative zeta potential values observed by other authors suggest that GR may exhibit a negative electrostatic charge, possibly due to anion adsorption or the nature of its functional groups [38]. These findings align with the previously reported FTIR results, which provide insight into the structural composition of the microspheres.

The results of the stability test for e-GR and e-GR-ZnPc are shown in Figure 6. The initial values obtained for both formulations indicated that the microspheres exhibited high stability under the studied conditions, with values of −80.6 ± 3.3 and −74.1 ± 5.6 mV for e-GR and e-GR-ZnPc, respectively. These values were maintained for 72 h, confirming their good short-term stability. Since gum rosin is highly hydrophobic, the use of 1 wt.% SDS was necessary to achieve homogeneous aqueous dispersions. Consequently, these highly negative zeta potential values and the excellent short-term stability are heavily driven by the adsorption of this anionic surfactant onto the particle surface. While the intrinsic carboxyl groups of the GR matrix contribute to the negative charge, the SDS coating primarily provides the strong electrostatic repulsion that maintains stable dispersion of the formulated microspheres. However, the zeta potential of the microspheres decreased at 120 h, potentially due to particle aggregation or coalescence. This decrease was gradual, and after 360 h of testing, the zeta potentials had decreased by approximately 70% to −26.5 ± 3.2 mV for e-GR and −24.1 ± 5.2 mV for e-GR-ZnPc. Notably, incorporating ZnPc did not significantly affect stability over the testing period. Do Reis et al. reported lower zeta potential values for ZnPc-encapsulated PLA/PVA nanoparticles, further highlighting the potential of GR in biomedical applications [30].

### 3.6. Release of the Active Compound

The release of phthalocyanines from e-GR-ZnPc microspheres was measured using a sodium chloride aqueous solution, which is commonly employed as an adequate simulant in saline or physiological conditions, as its concentration and properties closely resemble those found in body fluids. A 0.9 wt% concentration of NaCl equals an osmolarity highly similar to that of blood plasma [38]. Figure 7 shows the results obtained for the release test. The release experiments were conducted using independent vessels for each sampling time, thereby avoiding repeated sampling from the same vessel and the associated changes in the release medium volume and microspheres-to-medium ratio. This experimental design allowed the ZnPc concentration in the release medium to be determined at each time point without perturbing the system through previous sampling events.

As observed in Figure 7, the release profile of ZnPc from the GR microspheres exhibited a biphasic pattern, with an initial burst within the first 24 h, followed by sustained release that peaked after 72 h. These results reflect the fast release of ZnPc during the initial 24-h period, reaching a concentration of 0.438 ± 0.044 mg/100 g of sample. The maximum value was observed after 72 h (0.868 ± 0.071 mg/100 g of sample). This initial burst release is consistent with observations from other authors who studied various polymer-based drug delivery systems. For instance, PLGA nanoparticles were proposed for encapsulating ZnPc, achieving maximum drug release after 100 h in phosphate-buffered saline at 37.0 ± 0.5 °C with constant stirring [39]. Moreover, polymer-based nanocapsules containing ZnPc were investigated using the dialysis bag technique, demonstrating maximum drug release within 24 h in a sodium acetate-acetic acid buffer at pH 5.4 supplemented with 1 wt% SDS [40]. However, the sustained release observed in this study, likely governed by diffusion, differs from the release profile of PLGA nanoparticles, in which maximum release was achieved after 100 h. This difference highlights the influence of the polymer matrix on the release kinetics of encapsulated drugs. When compared to conventional biopolymeric carriers, such as PLGA or chitosan, the eGR-ZnPc system shows a distinct advantage in its processing simplicity and the use of a fully natural, low-cost matrix. While PLGA systems often require complex multi-step emulsion methods, the electrosprayed gum rosin microspheres achieve effective encapsulation and a favorable biphasic release profile in a single step. This highlights the significance of using unmodified gum rosin as a viable, highly sustainable alternative for photosensitizer delivery, matching the functional performance of established synthetic polymers without their associated manufacturing complexities. Following the peak release at 72 h, a decline in the measured ZnPc concentration was observed. This behavior may be related to the limited aqueous solubility of ZnPc and its tendency to self-associate in aqueous media. The planar structure of phthalocyanines favors π–π interactions, which can promote aggregation and reduce the fraction of ZnPc remaining in solution as measurable monomeric species [41]. Once released from the protective GR matrix, free ZnPc may gradually precipitate or adsorb onto container walls, thereby limiting its quantification in the aqueous medium. This highlights the effectiveness of the GR microspheres in initially dispersing the photosensitizer and points to the known solubility limits of free ZnPc in static aqueous environments [42,43]. However, these mechanisms should be considered as possible explanations rather than confirmed causes, as they were not directly assessed in the present study.

As detailed in Table 3, a quantitative benchmarking of the eGR-ZnPc system against other nanocarriers reveals distinct functional differences. While PLGA-PVA-ZnPc nanoparticles and micellar systems report higher Encapsulation Efficiencies (EE) (75%) and Loading Capacities (LC), they often rely on multi-step preparation methods (such as nanoprecipitation or dialysis) requiring surfactants or co-solvents. In contrast, the eGR-ZnPc formulation presents an EE of 18.1% and a highly dilute LC of 0.0026%. For powerful photosensitizers like ZnPc, a low LC is often advantageous to prevent intramolecular quenching and aggregation within the matrix, thereby maintaining a high quantum yield for singlet oxygen generation. The single-step electrospraying of gum rosin provides a functionally viable, surfactant-free alternative for photosensitizer delivery.

Furthermore, given the average diameter of the e-GR-ZnPc microspheres (20.9 µm), this formulation is specifically envisioned for localized drug-delivery applications, such as direct intra-tumoral injections, topical formulations, or depot implants. Unlike systemic intravenous administration, which requires nanoscale dimensions, localized delivery of microparticles is highly advantageous in photodynamic therapy, as it maximizes the photosensitizer concentration at the target site while minimizing off-target systemic distribution and subsequent generalized skin photosensitivity.

To further investigate the time-dependent ZnPc profile, the experimental data collected during the increasing phase (4–72 h) were fitted to zero-order, first-order, Higuchi, Korsmeyer–Peppas, and Weibull models (Table 4). The fit between experimental and model results was assessed using the coefficient of determination (R^2^) and the root mean square error (RMSE). Among the models evaluated, the Weibull model provided the best descriptive fit (R^2^ = 0.9611, RMSE = 5.123 × 10^−4^ mg/g), followed by the first-order model (R^2^ = 0.9584, RMSE = 5.302 × 10^−4^ mg/g). The Higuchi and Korsmeyer–Peppas models also showed reasonable agreement with the experimental data (R^2^ = 0.9522 and R^2^ = 0.9473 respectively), whereas the zero-order model yielded a comparatively lower correlation (R^2^ = 0.8904).

The fitted Weibull parameters were Q∞ = 0.009982 mg/g, α = 1.163 × 10^5^ s, and β = 0.8696. In this model, Q∞ represents the asymptotic value predicted by the fitted curve, α is the scale parameter associated with the characteristic time of the process, and β is the shape parameter governing the curve profile. Specifically, α corresponds to the time at which the predicted response reaches approximately 63.2% of Q∞, whereas β < 1 describes a decelerating increase in the fitted response. Nevertheless, these parameters must be interpreted cautiously. Estimation of characteristic time scales in diffusion-controlled release systems can be sensitive to the duration of the experimental observation window, particularly when the available data do not adequately characterize the asymptotic regime. In the present study, the Weibull model was fitted only to the increasing phase of the measured concentration profile (4–72 h); consequently, Q∞ should be regarded as a model-derived asymptotic value rather than an experimentally established total amount released.

Overall, the kinetic analysis provides a descriptive comparison of mathematical models applied to the increasing portion of the detected ZnPc concentration profile. Although the Weibull model provided the best descriptive fit among all models evaluated, and the Higuchi and other models also showed reasonable agreement with the experimental data, the fit alone does not establish a diffusion-controlled release mechanism. Furthermore, the decrease in the detected ZnPc concentration after 72 h cannot be explained conclusively by the present kinetic analysis. Further experiments, including a mass-balance assessment and characterization of ZnPc in both the release medium and the residual microspheres, would be required to clarify the processes responsible for this decrease.

## 4. Conclusions

The electrospraying method was successfully used to integrate ZnPc into GR microspheres. This encapsulation strategy has shown promise in enhancing ZnPc bioavailability, stability, and targeted transport. FESEM evaluation revealed a significant increase in microsphere size; this substantial growth is driven not solely by the physical mass of ZnPc, but by altered microscopic electrospraying dynamics and early nucleation induced by the photosensitizer. While FESEM images showed clustering, zeta potential measurements confirmed that the microspheres maintained strong electrostatic repulsion and dispersed as independent entities in aqueous media when formulated with a surfactant (SDS), demonstrating that the observed aggregation is primarily caused by loss of solvent during drying.

The structural integration of ZnPc into the GR matrix was unequivocally confirmed. Difference FTIR spectra revealed distinct interaction bands, while 1H-NMR demonstrated significant shielding and deshielding effects exerted by the highly aromatic ZnPc macrocycle on the rosin protons. Together, these techniques confirm that the system is not a simple physical mixture but rather involves strong intermolecular interactions (electrostatic or hydrogen-bonding).

The release profile of ZnPc from the GR microspheres exhibited a biphasic pattern, with an initial burst followed by sustained release that peaked at 72 h. The apparent decline in the measured concentration thereafter highlights the extreme hydrophobicity of the free photosensitizer, leading to its eventual precipitation or adsorption onto the container walls in a closed, static system.

While this study successfully establishes the physicochemical feasibility, morphological stability, and release behavior of the eGR-ZnPc delivery system, its ultimate application in photodynamic therapy requires biological validation. Therefore, future studies will focus on evaluating the specific photodynamic activity of the released ZnPc, including singlet oxygen generation efficiency and in vitro cytotoxicity, to enable the translation of this natural matrix into clinical applications.

## Figures and Tables

**Figure 1:**
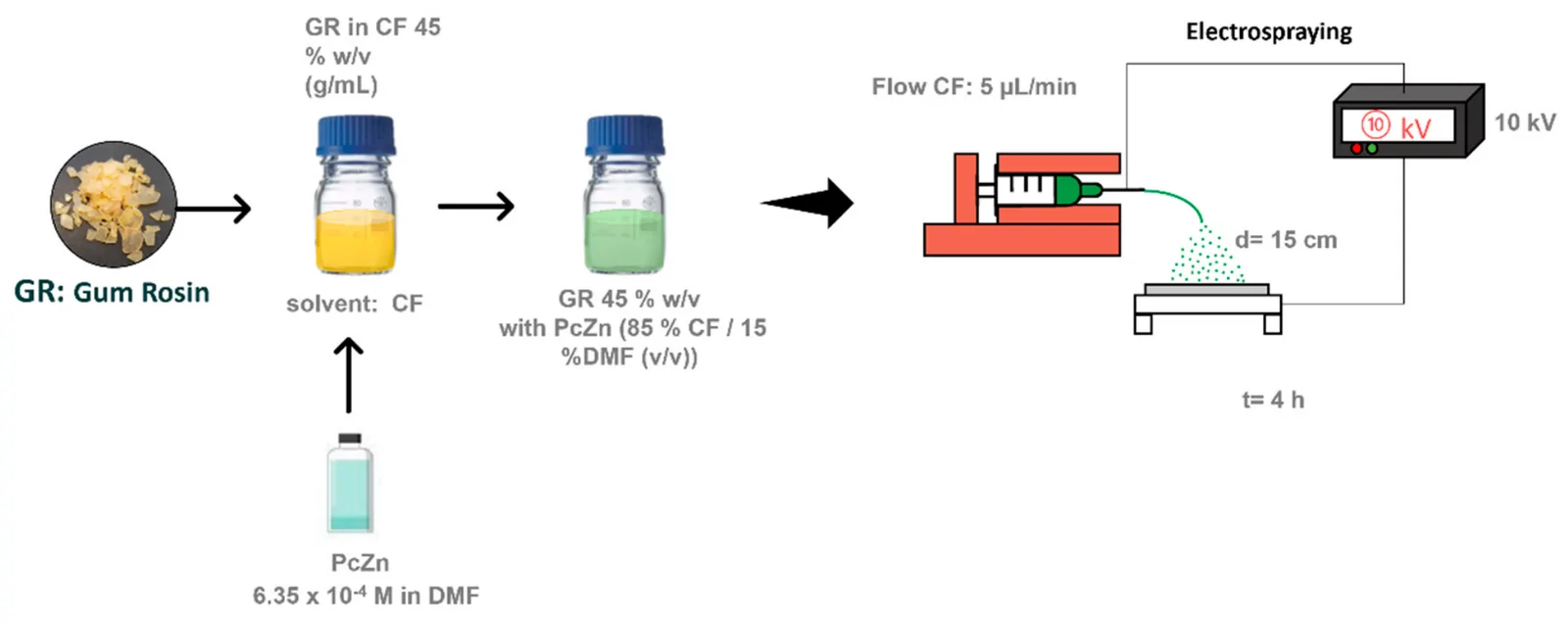
Electrospraying process scheme of gum rosin solution with zinc phthalocyanine.

**Figure 2:**
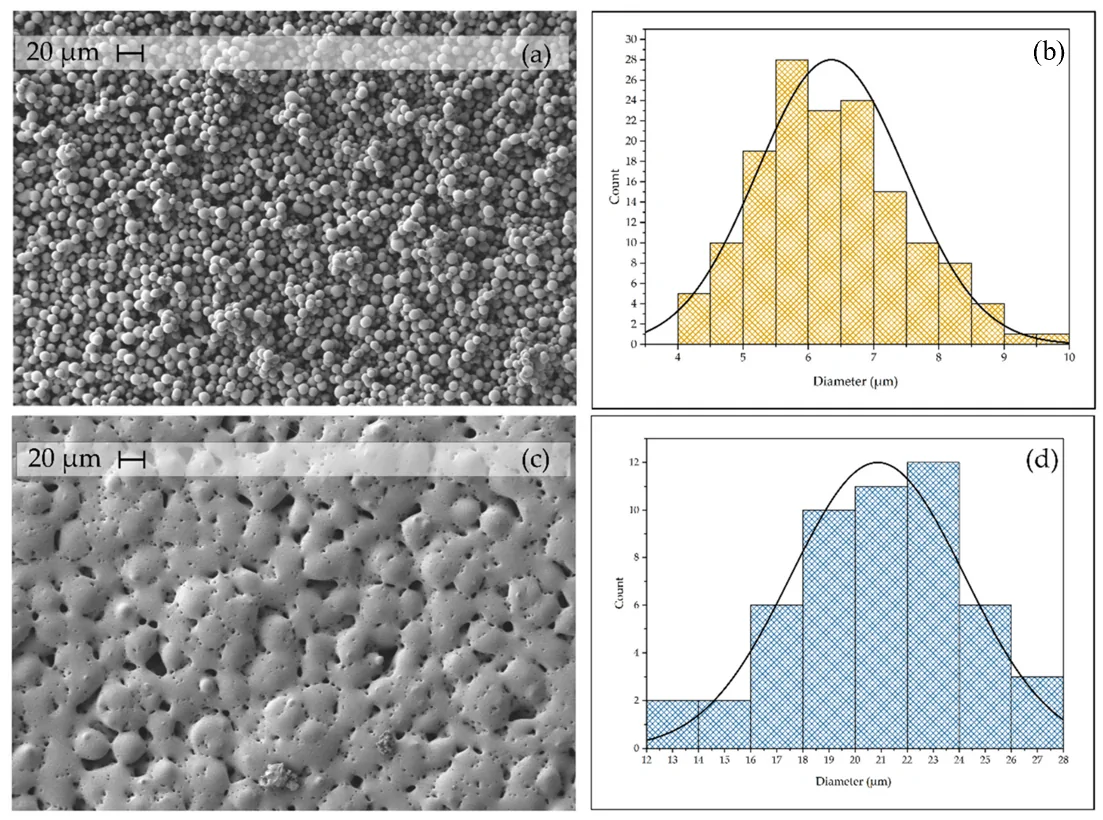
FESEM images of e-GR (**a**) and e-GR-ZnPc (**c**), along with histograms of their diameter distributions, e-GR (**b**) and e-GR-ZnPc (**d**).

**Figure 3:**
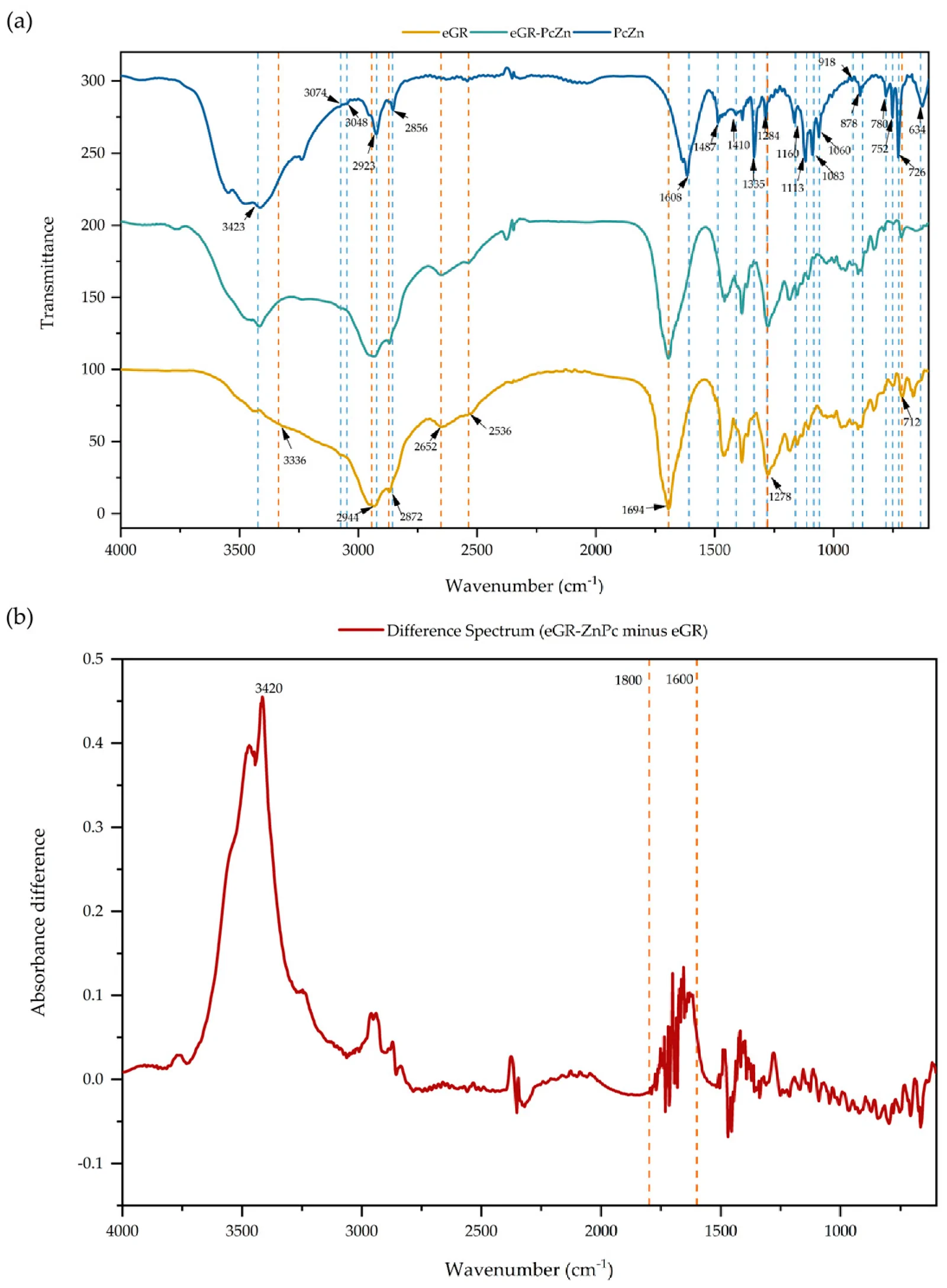
(**a**) FTIR spectra for gum rosin microspheres (e-GR) (orange), zinc phthalocyanine (ZnPc) (blue), and gum rosin microspheres charged with ZnPc (eGR-ZnPc) (green). (**b**) Difference FTIR spectrum obtained by subtracting the e-GR spectrum from eGR-ZnPc.

**Figure 4:**
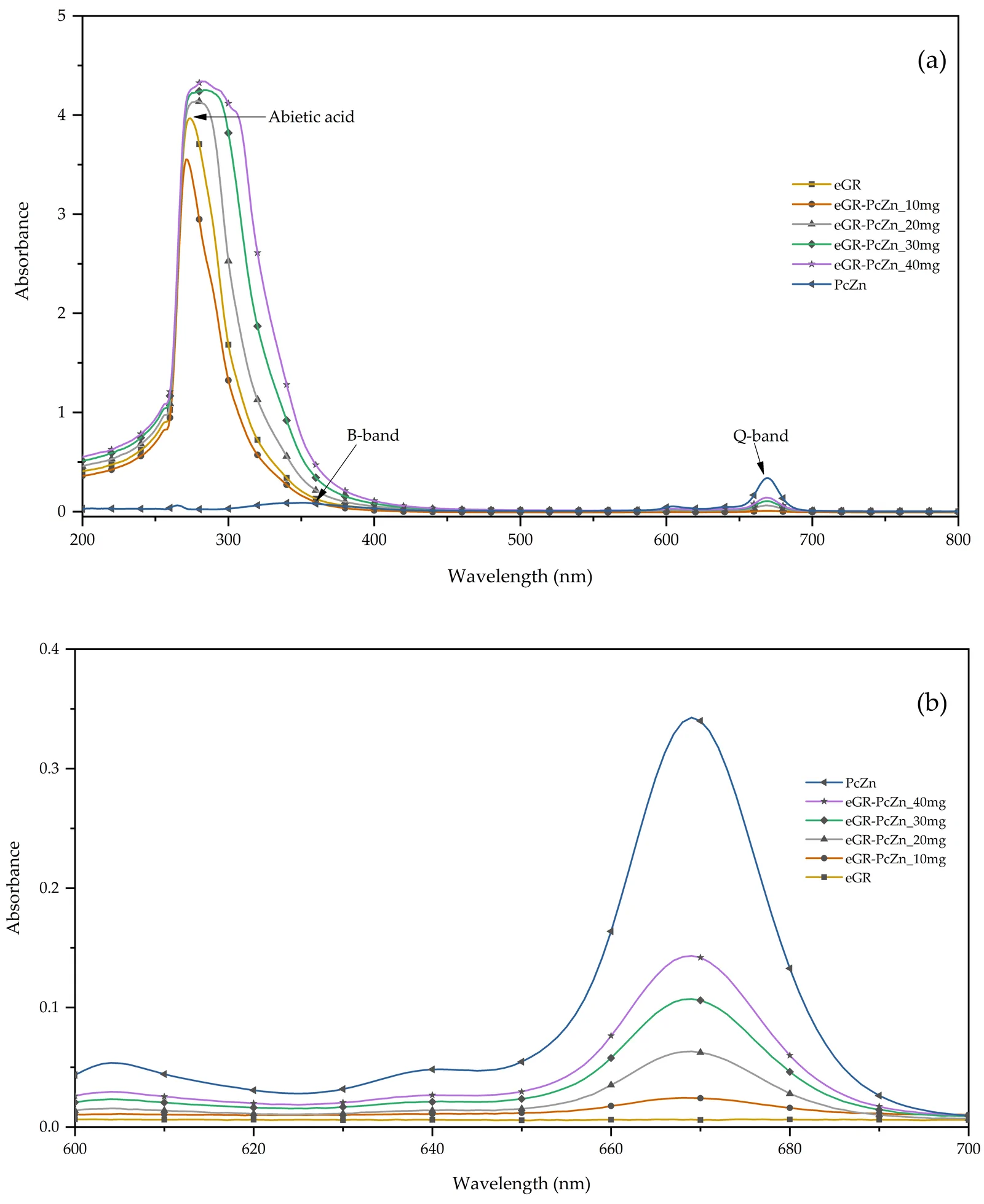
UV-Vis spectra for gum rosin microspheres (e-GR), zinc phthalocyanine (ZnPc), and gum rosin microspheres charged with ZnPc (eGR-ZnPc). (**a**) Full graph; (**b**) region of the amplified spectrum between 600 and 700 nm.

**Figure 5:**
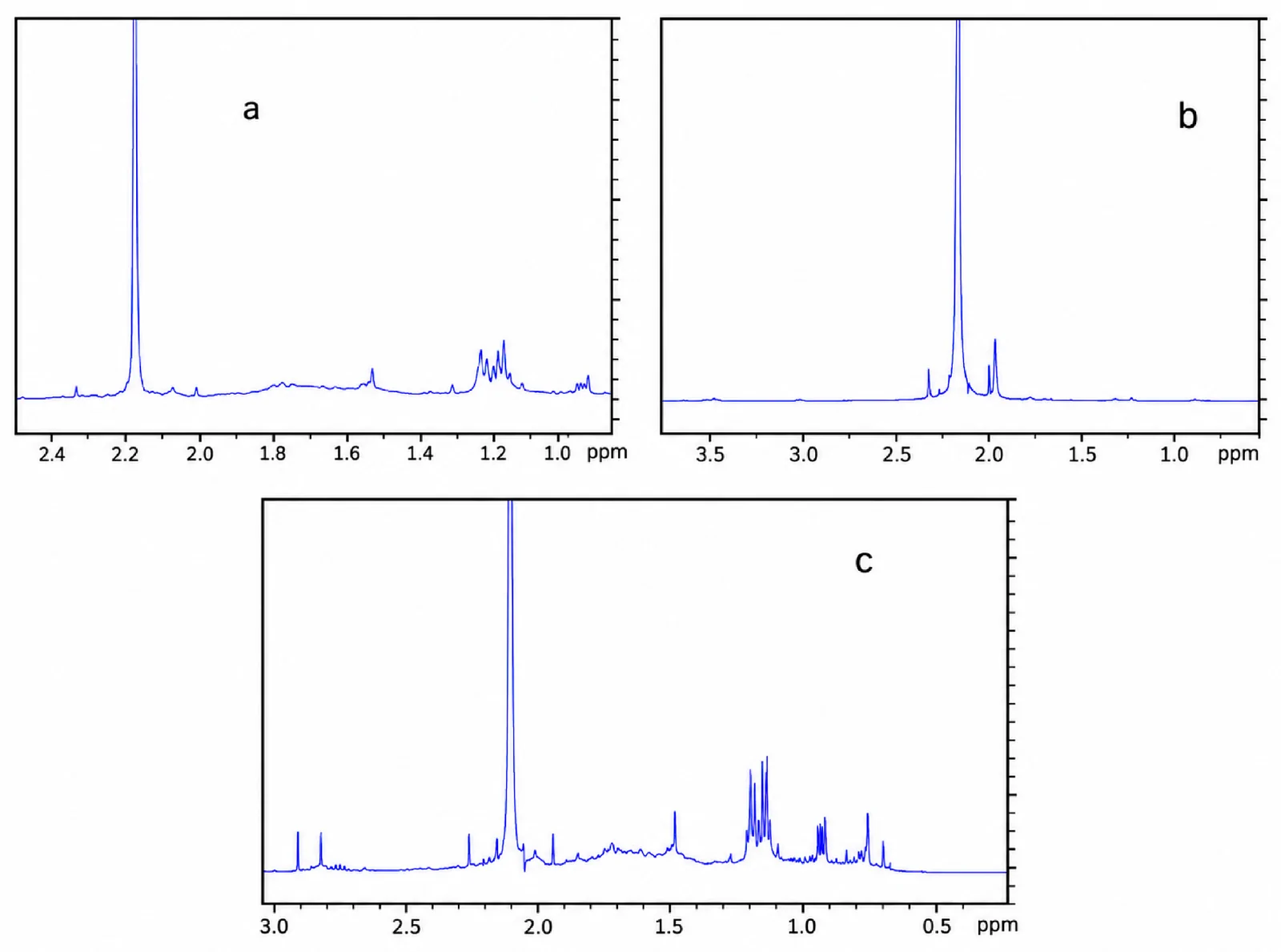
NMR spectra of (**a**) e-GR, (**b**) ZnPc, and (**c**) e-GR-ZnPc.

**Figure 6:**
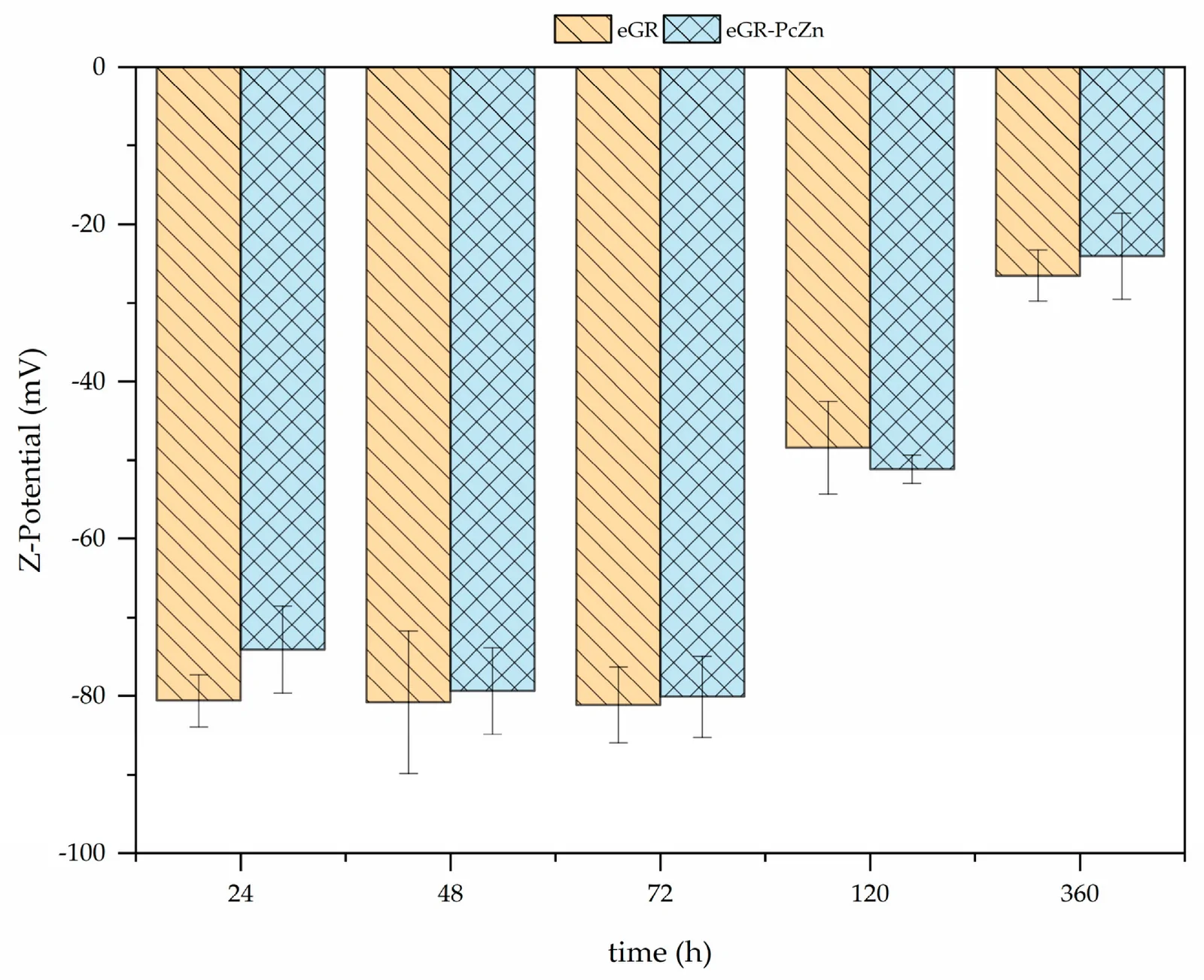
Z-potential values obtained by different microspheres stored under refrigerated conditions for 360 h. (mean ± SD, n = 3).

**Figure 7:**
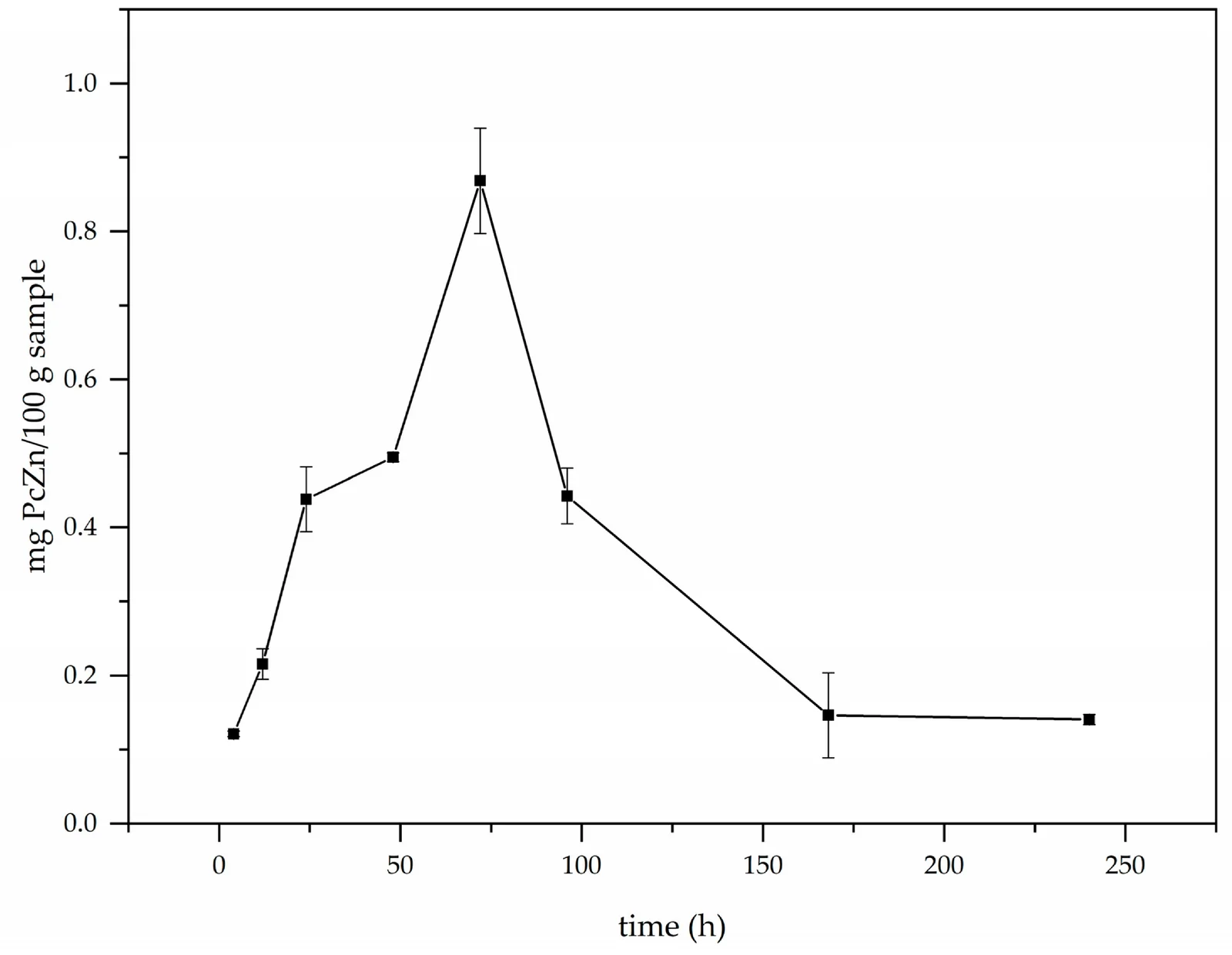
Kinetic release of eGR-ZnPc into 0.9%NaCl under controlled conditions (mean ± SD, n = 3).

**Table 1:** Comparative analysis of previous GR-based delivery approaches versus the proposed eGR-ZnPc formulation.

GR-Based Delivery System	Preparation Method	Limitations of Previous Approaches	Advantages of the Proposed eGR-ZnPc Formulation
GR Macroscopic Matrices/Films	Melt extrusion, solvent casting.	Requires high temperatures (risk of thermal degradation for sensitive drugs); yields a low surface-area-to-volume ratio, limited release control; irregular distribution.	Room-temperature processing: Preserves the chemical stability of the photosensitizer [8]
GR Micro/Nanoparticles	Emulsion-solvent evaporation, nanoprecipitation	Multi-step procedures require the addition of surfactants or stabilizers (e.g., PVA, Tween), which may induce toxicity or interact with the active compound.	Surfactant-free & Single-step: Simplifies manufacturing, prevents secondary chemical interactions, and yields high purity [9]
eGR-ZnPc Microspheres (This work)	Electrospraying	—	Tunable micro-morphology: High surface area enables a highly controlled, biphasic release profile ideal for photodynamic therapy.

**Table 2:** Concentration of ZnPc deposited on e-GR microspheres for different masses of microspheres.

Mass (mg)	Sample Label	Absorbance	Zinc Phthalocyanine Concentration (M)
10	eGR-ZnPc_10 mg	0.024327	8.63 × 10^−8^
20	eGR-ZnPc_20 mg	0.063250	2.24 × 10^−7^
30	eGR-ZnPc_30 mg	0.107144	3.80 × 10^−7^
40	eGR-ZnPc_40 mg	0.143213	5.08 × 10^−7^

**Table 3:** Quantitative comparison of the eGR-ZnPc system with conventional biopolymeric carriers.

Delivery System	Matrix Material	Preparation Method	Encapsulation Efficiency (EE %)	Loading Capacity (LC %)	Reference
eGR-ZnPc	Gum Rosin	Single-step electrospraying	18.1	0.0026	This work
PLGA-PVA-ZnPc	Synthetic polymer	Emulsion solvent evaporation	75.67	~3.0	[40]
SDC/TPGS Micelles	Surfactant mixture	Dialysis	92.13	Not reported	[43]

**Table 4:** Kinetic model fitting to the experimentally detected ZnPc content (4–72 h).

Kinetic Model	Fitted Equation	R^2^	RMSE (mg/g)
Zero-order	Q_t_ = Q_0_ + k_0_t	0.8904	8.602 × 10^−4^
First-order	Q_t_ = Q_∞_(1 − e^−kt^)	0.9584	5.302 × 10^−4^
Higuchi	Q_t_ = b + K_H_ t^0.5^	0.9522	5.678 × 10^−4^
Korsmeyer–Peppas	Q_t_ = Kt^n^	0.9473	5.965 × 10^−4^
Weibull	Q_t_ = Q_∞_[1 − e^−(t/α)β^]	0.9611	5.123 × 10^−4^

## Data Availability

The raw data supporting the conclusions of this article will be made available by the authors on request.
